# Metabolic Health in Obese Subjects—Is There a Link to Lactoferrin and Lactoferrin Receptor-Related Gene Polymorphisms?

**DOI:** 10.3390/nu12092843

**Published:** 2020-09-17

**Authors:** Małgorzata Jamka, Nina Kaczmarek, Edyta Mądry, Patrycja Krzyżanowska-Jankowska, Joanna Bajerska, Matylda Kręgielska-Narożna, Paweł Bogdański, Jarosław Walkowiak

**Affiliations:** 1 Department of Pediatric Gastroenterology and Metabolic Diseases, Poznan University of Medical Sciences, Szpitalna Str. 27/33, 60-572 Poznań, Poland; nkaczmarek@ump.edu.pl (N.K.); pkrzyzanowska@ump.edu.pl (P.K.-J.); jarwalk@ump.edu.pl (J.W.); 2 Department of Physiology, Poznan University of Medical Sciences, Święcickiego Str. 6, 60-781 Poznań, Poland; emadry@ump.edu.pl; 3 Institute of Human Nutrition and Dietetics, Poznan University of Life Sciences, Wojska Polskiego Str. 31, 60-624 Poznań, Poland; joanna.bajerska@up.poznan.pl; 4 Department of Treatment of Obesity, Metabolic Disorders and Clinical Dietetics, Poznan University of Medical Sciences, Szamarzewskiego Str. 84, 60-569 Poznań, Poland; matkreg@ump.edu.pl (M.K.-N.); pbogdanski@ump.edu.pl (P.B.)

**Keywords:** lactoferrin, low-density lipoprotein receptor-related protein 1, low-density lipoprotein receptor-related protein 2, obesity, single nucleotide polymorphism

## Abstract

This study aimed to evaluate the association of genetic variants in lactoferrin (*LTF*) metabolism-related genes with the prevalence of metabolically healthy obesity (MHO) and metabolically unhealthy obesity (MUHO). In total, 161 MHO and 291 MUHO subjects were recruited to the study. The following polymorphisms were genotyped: low-density lipoprotein receptor-related protein (*LRP*) 2 rs2544390, *LRP1* rs4759277, *LRP1* rs1799986, *LTF* rs1126477, *LTF* rs2239692 and *LTF* rs1126478. We found significant differences in the genotype frequencies of *LTF* rs2239692 between MHO and MUHO subjects, with the CT variant associated with lower odds of developing metabolic syndrome than the TT variant. In the total population, significant differences in body weight and waist circumference (WC) were identified between *LTF* rs1126477 gene variants. A similar association with WC was observed in MUHO subjects, while significant differences in body mass index and low-density lipoprotein cholesterol levels were discovered between *LTF* rs1126477 gene variants in MHO subjects. Besides, there were significant differences in diastolic blood pressure between *LRP1* rs1799986 gene variants in MUHO subjects, as well as in WC and high-density lipoprotein cholesterol levels between *LRP1* rs4759277 gene variants in MHO subjects. In conclusion, selected lactoferrin and lactoferrin receptor-related gene variants may be associated with the prevalence of metabolically healthy or metabolically unhealthy obesity.

## 1. Introduction

Obesity is an important global health problem. According to the World Health Organization (WHO), obesity prevalence rates tripled between 1975 and 2016, and currently, around 650 million people worldwide (15% of women and 11% of men aged ≥18 years) suffer from excessive body weight [1]. Obesity is not only a cosmetic defect but can also cause a variety of metabolic abnormalities, leading to the deterioration of health and quality of life. It is well known that excessive body weight is associated with insulin resistance and hyperinsulinemia. Consequently, obesity is recognised as an important risk factor in the pathogenesis of type 2 diabetes mellitus [2]. Furthermore, obesity also increases the risk of developing cardiovascular diseases, hypertension, dyslipidaemia and several other abnormalities associated with the development of metabolic syndrome [3].

Metabolic syndrome represents several cardiovascular risk factors associated with obesity, such as disturbed glucose and insulin homeostasis, atherogenic dyslipidaemia and arterial hypertension. These components of metabolic syndrome are also complemented with chronic low-grade inflammation, coagulopathy, endothelial dysfunction and oxidative stress [4]. The prevalence of metabolically unhealthy obesity is estimated to be around 75%, while approximately 25% of obese subjects are metabolically healthy. Unfortunately, factors associated with healthy and unhealthy obesity still remain unclear. Nevertheless, early identification of subjects at high risk of developing metabolic abnormalities might allow appropriate preventive and curative measures to be taken [5].

The metabolic syndrome usually develops as a consequence of increased energy intake and low level of physical activity, with other important factors including diet composition, aging population and genetic background [6]. Previously, several genes have been identified as potentially associated with different features of the metabolic syndrome, such as transcription factor 7-like 2 (*TCFL2*), fat mass and obesity gene (*FTO*), adenylate cyclase type 5 (*ADCY5*), fatty acid desaturase 1 (*FADS1*), GLI-similar 3 (*GLIS3*), insulin-like growth factor 1 (*IGF1*) and peroxisome proliferator-activated receptor-gamma (*PPARγ*) [7,8,9].

It has been hypothesised that variants in lactoferrin (*LTF*) and lactoferrin receptor-related genes may also play an important role in the development of metabolic abnormalities in obese subjects. Previously, *LTF* gene polymorphisms were reported to be associated with lactoferrin levels and coronary artery stenosis [10]. Moreover, *LTF* rs1126477 and rs1126478 polymorphisms were reported to be associated with high-density lipoprotein cholesterol (HDL-C) and triglycerides (TG) levels in subjects with impaired glucose tolerance [11]. Interestingly, in children, three single nucleotide polymorphisms (SNPs)—rs1126478, rs34827868 and rs1042073—in the *LTF* gene had a minor allele associated with increased HDL-C concentrations and three others (rs4637321, rs2239692 and rs10865941) were related with decreased fasting glucose levels, increased blood pressure, and higher levels of free fatty acids. However, these associations did not remain significant after correction for multiple testing [12]. Another study demonstrated that the hypolipidemic effect of lactoferrin is dependent on the selective binding of this protein to the low-density lipoprotein receptor-related protein 1 (LRP1). It was assumed that modifications of the lactoferrin molecule affect the interaction with LRP1 receptors, thus influencing the level of lipids and the rate of their removal from the circulation. Indeed, the Arg-rich sequence of the N-terminus of the lactoferrin resembles the structure of apolipoprotein E recognised by LRP1 [13]. In addition, some gene variants in *LRP1* rs4759277 are associated with insulin concentrations and homeostasis model assessment of insulin resistance (HOMA-IR) [7]. However, whether the above polymorphisms in lactoferrin-related genes influence the development of metabolic syndrome in obese subjects remains unclear.

Therefore, the study aimed to assess the prevalence of selected *LTF* and lactoferrin receptor gene polymorphisms in metabolically healthy obese (MHO) and metabolically unhealthy obese (MUHO) subjects, examining the impact of analysed gene polymorphisms on individual components of the metabolic syndrome.

## 2. Materials and Methods

### 2.1. Study Population

Obese men and women were recruited to the study at the Poznan University of Medical Sciences, Poland. During the admission process, subjects received information about the study, its aim, possible benefits and risks. Study participants were informed that participation in the study was voluntary and that they may refuse to participate or discontinuing participation at any time without giving reasons. Written informed consent was obtained from all participants.

In total, 452 obese subjects were included in the study and were divided into two groups: MHO and MUHO group. The International Diabetes Foundation criteria were used to identify metabolic syndrome [14,15]. The primary inclusion criteria were as follows: body mass index (BMI) ≥ 30 kg/m^2^ or waist circumference (WC) ≥ 80 cm for women, and ≥94 cm for men. In addition, during dividing the study population into two groups, the following inclusion criteria were used: (1) MHO: less than two of the following disorders, (2) MUHO: at least two of the following disorders:

− systolic blood pressure (SBP) ≥ 130 mmHg and/or diastolic blood pressure (DBP) ≥ 85 mmHg and/or antihypertensive therapy;
− TG levels ≥ 150 mg/dL (1.7 mmol/L) and/or specific treatment for this lipid abnormality;
− fasting glucose levels ≥ 100 mg/L (5.6 mmol/L) and/or previously diagnosed type 2 diabetes;
− HDL-C levels < 40 mg/L (1.03 mmol/L) in men, <50 mg/dL (1.29 mmol/L) in women and/or specific treatment for this lipid abnormality [4,14].

The exclusion criteria were as follows:

− cancer diagnosis in the last 5 years;
− general poor health status;
− pregnant and breastfeeding women.

This study was conducted according to the Declaration of Helsinki. The study protocol was approved by the Poznan University of Medical Sciences Bioethical Committee (refs. 984/17, 1161/19).

### 2.2. Assessment of Anthropometric Parameters

The following anthropometric parameters were assessed in this study: body weight, body height and WC. During anthropometric measurements, all participants wore light clothes and were barefoot. Body height was measured in the standing position and rounded up to the nearest 0.5 cm. Body weight and body height were measured by a calibrated electronic scale with a stadiometer. WC was assessed on the bare skin between the lowest rib and the iliac crest, during minimal respiration. In this study, the WHO criteria were used to defined abdominal obesity (WC ≥ 94 cm in men and ≥ 80 cm in women) [15], measured using a standard tape measure. BMI was calculated based on body weight and body height, defined as body weight in kilograms divided by body height in meters squared and classified according to the WHO criteria [16].

### 2.3. Blood Pressure

Blood pressure was measured according to the European Society of Hypertension guidelines. SBP and DBP were measured on the arm at heart level and were expressed by three measurements. Normal blood pressure is defined as SBP < 130 mmHg and DBP < 85 mmHg, while hypertension is defined as SBP ≥ 140 mmHg or DBP ≥ 90 mmHg for most adults [17].

### 2.4. Blood Collection and Biochemical Measurements

Blood samples were taken fasting by registered staff nurses. All biochemical parameters were measured using standardised laboratory methods, including fasting plasma glucose concentrations, serum concentrations of insulin, total cholesterol (TC), HDL-C, low-density lipoprotein cholesterol (LDL-C), TG and C-reactive protein (CRP; data available for 450 subjects; for 258 subjects exact CRP levels were available, for 192 subjects CRP levels were measured by standard methods and the analyser was not able to detect values < 4 mg/L).

### 2.5. Genotyping

Genomic DNA was isolated from EDTA anticoagulated blood according to the membrane-based DNA extraction protocol (Blood Mini, A&A Biotechnology, Poland, or equivalent). In short, defrosted blood samples of 1 mL were mixed with 500 μL LE and then centrifugated at 10,000–15,000 revolutions per minute (rpm) for 3 min. Then, the supernatant was discarded, 100 µL of Tris buffer was added and cells were resuspended by pipetting. For the prepared samples 20 μL protease K and 200 μL buffer LT were added. After incubation at 37 °C for 20 min, 20 s of the vortex, 1 min of centrifugation at 10,000–15,000 rpm and discarded the filtrates, 500 μL of A1 wash solution was added. The mixture was centrifuged at 10,000–15,000 rpm for 1 min, and another 400 μL of A1 wash solution was added. After centrifuging at 10,000–15,000 rpm for 2 min, 100 μL of Tris elution buffer heated to 75 °C was added. The mixture was incubated for 5 min at room temperature and centrifuged for one minute at 10,000–15,000 rpm. The yielded DNA concentrations were measured using a NanoDrop™ One Spectrophotometer (ThermoScientific, Wilmington, NC, USA). SNPs in *LTF* rs1126477, *LTF* rs2239692, *LTF* rs1126478, low-density lipoprotein receptor-related protein 2 (*LRP2*) rs2544390, *LRP1* rs4759277, and *LRP1* rs1799986, genes were genotyped with TaqMan allelic discrimination assays (Applied Biosystem, Foster City, NC, USA): rs2544390: C___8822318_10, rs4759277: C___31186847_10, rs1799986: C___1955081_10, rs1126477: C___9698511_10; rs2239692: C___2610649_10; rs1126478: C___9698521_10) using Bio-Rad CFX96™ Real-Time PCR system (Hercules, CA, USA). The PCR reactions (10 μL) contained: (1) TaqPath ProAmp Master Mix—5 μL, (2) TaqMan Genotyping Assay (containing sequence-specific forward and reverse primers) —0.5 μL, (3) DNA—4.5 μL, (4) Nuclease free water—10 μL. The 96-well plates were used. In 95 wells, genomic DNA was disposed of. Each plate consisted of one negative control. After the addition of the TaqPath ProAmp Master Mix, the plate was covered with the PCR plate sealer and briefly centrifuged in the plate centrifuge. Amplification conditions were as follows: (1) Pre-read run—30 s—60 °C—hold, (2) Enzyme activation—5 min—95 °C—hold, (3) Denaturation—15 s—95 °C—40 cycles, (4) Anneal/extend—1 min—60 °C—40 cycles, (5) Post-read run—30 s—60 °C—hold.

### 2.6. Statistical Analysis

The STATISTICA 12.0 PL (StatSoft, Tulsa, OK, USA) and PQStat (PQStat Software, Poznań, Poland) software (α = 0.05) were used for the statistical analysis. A two-sided *p*-value < 0.05 was considered as statistically significant. The overall characteristics of subjects were expressed as medians and interquartile ranges (IQRs), means and standard deviations (SDs) or as frequencies and percentages. The normality of the distribution of the variables was verified using the Shapiro–Wilk test. Allele and genotype frequencies of the analysed polymorphisms were tested for consistency with Hardy-Weinberg equilibrium using exact tests. De Finetti diagrams with Hardy-Weinberg parabola were generated using the online programme tool [18]. Allele frequency differences were assessed by the Chi^2^ test and genotype differences by Armitage’s trend test [18]. Quantitative phenotypic traits were determined using Mann–Whitney U and Kruskal–Wallis tests. Post-hoc analysis was performed for pairwise comparisons of subgroups using Dunn’s test with Bonferroni correction. Contingency tables were used to assess relationships between categorical variables.

## 3. Results

### 3.1. Study Cohort

Table 1 summarises the clinical characteristics of the study population. In total, 452 subjects (79.4% of women) with a median age of 61 (55–65) years were recruited into the study. The median BMI and WC of the study population were 31.73 (29.93–35.08) kg/m^2^ and 104.5 (97.0–110.0) cm.

### 3.2. Comparison of MHO and MUHO Subjects

Based on the International Diabetes Federation criteria, the study population was divided into two groups: MHO (*n* = 161) and MUHO (*n* = 291), as shown in Table 2. As expected, in comparison to the MUHO group, MHO subjects were significantly younger and displayed significantly lower WC, TC, TG and glucose levels, as well as SBP and DBP, whereas serum HDL-C concentrations were significantly higher. In addition, in the MUHO group significantly more subjects received pharmacological treatment (*p* = 0.0054), while in the MHO group statistically significant higher percentage of subjects had CRP values < 4 mg/L than in the MUHO group (*p* = 0.0120). When pharmacologically treated, subjects were excluded from the analysis similar differences between groups were observed. However, there were no significant differences in TC levels and CRP levels between the MHO group and the MUHO group (see Supplementary Table S1).

### 3.3. Distribution of Genotypes in Analysed Gene Polymorphisms in MHO and MUHO Subjects

Table 3 compares the allele and genotype frequencies of the tested SNPs between MHO and MUHO subjects. All allele distributions were in Hardy-Weinberg equilibrium (*p* > 0.05) and consistent with the frequencies published in the 1000Genomes database. There were significant differences in genotype frequencies of *LTF* rs2239692 (*p* = 0.0220) between the MHO group and the MUHO group. Similarly, significant differences in genotype frequencies of *LTF* rs2239692 between groups were observed when the analysis was restricted to non-pharmacologically treated subjects (*p* = 0.0113; see Supplementary Table S2).

### 3.4. Association of Genetic Variants with Prelevance of Metabolic Syndrome

Table 4 shows odds ratios (OR) and 95% confidence interval (CI) for the associations between analysed gene polymorphisms and the prevalence of metabolic abnormalities in obese subjects. Overall, the presence of the CT variant compared to the TT variant of *LTF* rs2239692 significantly decreased the odds of developing metabolic syndrome in obese subjects (OR = 0.53, 95% CI = 0.31–0.91, *p* = 0.0204). Other analysed SNPs did not significantly influence the risk of developing metabolic abnormalities (*p* > 0.05).

### 3.5. Impact of Analysed Gene Polymorphisms on Individual Components of Metabolic Syndrome

The effects of the analysed gene polymorphisms on the individual components of metabolic syndrome and other analysis parameters are presented in Supplementary Tables S3–S20. In the total population, significant differences in body weight (CC vs. CT vs. TT: 85.6 (78.6–96.5) vs. 83.0 (76.5–92.7) vs. 89.9 (79.0–100.8) kg, *p* = 0.0422) and WC (CC vs. CT vs. TT: 105.0 (97.4–112.0) vs. 103.0 (97.0–109.5) vs. 108.7 (103.5–117.0) cm, *p* = 0.0304) were observed between *LTF* rs1126477 gene variants. A similar association between WC and the *LTF* rs1126477 polymorphism was seen in the MUHO group (CC vs. CT vs. TT: 106.0 (99.0–114.0) vs. 104.0 (98.0–109.0) vs. 109.5 (104.0–120.0) cm, *p* = 0.0437), whereas significant differences in BMI (CC vs. CT vs. TT: 32.10 (30.04–34.45) vs. 30.78 (29.89–32.91) vs. 36.77 (33.36–37.30) kg/m^2^, *p* = 0.0349)and LDL-C levels (CC vs. CT vs. TT: 114 (92–136) vs. 121 (99–154) vs. 154 (154–174) mg/dL, *p* = 0.0267) were noticed between *LTF* rs1126477 gene variants in MHO subjects. In addition, we found significant differences in DBP between *LRP1* rs1799986 gene variants in MUHO subjects (CC vs. CT vs. TT: 87 (81–93) vs. 86 (79–94) vs. 100 (97–107) mmHg, *p* = 0.0173) as well as in WC (AA vs. AC vs. CC: MHO–100.0 (94.0–104.0) vs. 103.0 (95.0–110.0) vs. 105.0 (97.0–113.0) cm, *p* = 0.0209) and HDL-C levels (AA vs. AC vs. CC: MHO–66 (60–74) vs. 60 (52–68) vs. 59 (56–69) mg/dL, *p* = 0.0336) between *LRP1* rs4759277 gene variants in the MHO group. The post-hoc analyses revealed no significant differences in body weight in the total population. Similarly, there were no significant differences in WC in MUHO subjects and LDL-C levels in the MHO group between CC and CT, CC and TT as well as CT and TT genotypes of the *LTF* rs1126477 polymorphism (*p* > 0.05; data not shown). However, there were significant differences between the CT and TT variants of the *LTF* rs1126477 gene polymorphism in WC in the total population (*p* = 0.0463; data not shown) and in BMI in the MHO group (*p* = 0.0333; data not shown). We noted that the CT variant of *LTF* rs1126477 was associated with lower WC and BMI. Besides, significant differences in DBP between the CC and TT variants (*p* = 0.0208; data not shown) as well as the CT and TT variants (*p* = 0.0133; data not shown) of the *LRP1* rs1799986 polymorphism were found in MUHO subjects. We concluded that the TT variant was associated with a higher DBP. Moreover, in the MHO group, significant differences were detected in WC (*p* = 0.0170; data not shown) between the AA and CC gene variants and in HDL-C levels (*p* = 0.0275; data not shown) between the AA and AC gene variants of the *LRP1* rs4759277 polymorphism. The AA gene variant was related to lower WC and higher HDL-C levels.

Taking into account the possibility of the interaction between analysed genes polymorphisms, we also assessed the combined effect of the protective or risk genotypes of different SNPs on health status and biochemical parameters. Therefore, we divided the study population into two subgroups according to the number of protective or risk genotypes: <2 or ≥2. Based on the above presented results the following genotypes were classified as protective: *LTF* rs2239692–CT, *LTF* rs1126477–CT, *LRP1* rs1799986–CT or CC, *LRP1* rs4759277–AA and the following as risk: *LTF* rs2239692–TT, *LTF* rs1126477–TT, *LRP1* rs1799986–TT, *LRP1* rs4759277–AC or CC. We did not observe any differences between the percentage of subjects who had at least or less than two protective or risk genotypes between MHO and MUHO subjects (see Supplementary Table S21). Nevertheless, in the total population, we showed that the subjects who had at least two protective genotypes had significantly lower body weight (<2 vs. ≥2 protective genotypes: 86.5 (78.4–98.0) vs. 84.0 (77.1–93.2) kg, *p* = 0.0497) and significantly higher percentage of subjects had CRP values < 4 mg/L (<2 vs. ≥2 protective genotypes: 63.7% vs. 72.8%, *p* = 0.0396). In the MUHO group, we also found that the subjects who had at least two protective genotypes presented lower body weight (<2 vs. ≥2 protective genotypes: 87.1 (78.0–99.0) vs. 84.9 (77.2–92.6) kg, *p* = 0.0497), WC (<2 vs. ≥2 protective genotypes: 106.5 (100.0–114.0) vs. 104.0 (97.5–110.0) cm, *p* = 0.0443) and DBP (<2 vs. ≥2 protective genotypes: 88 (82–96) vs. 86 (79–92) mmHg, *p* = 0.0113; see Supplementary Table S22). However, in the MUHO group, we did not observe any differences in analysed parameters between subjects who had at least two or less than two risk genotypes. Nevertheless, in the total population and the MHO group, we demonstrated that subjects who had at least two risk genotypes had significantly lower HDL-C levels (<2 vs. ≥2 risk genotypes: total population: 58 (49–68) vs. 55 (47–63) mg/dL, *p* = 0.0328, MHO group: 65 (59–74) vs. 59 (53–66) mg/dL, *p* = 0.0035) compared to subjects with less than two risk genotypes (see Supplementary Table S23).

## 4. Discussion

One of the key findings of this research were differences in genotype frequencies of the *LTF* rs2239692 between MHO and MUHO subjects. Moreover, we found that the CT variant compared to the TT variant of this polymorphism was associated with lower odds of developing metabolic syndrome. Furthermore, we demonstrated several associations between analysed gene polymorphisms and individual components of the metabolic syndrome. To the best of our knowledge, no studies have yet compared the prevalence of selected *LTF* and lactoferrin receptor genes polymorphisms in MHO and MUHO subjects.

In 1980, Andres first suggested that obese subjects should be classified into two groups: MHO and MUHO [19], with the MHO group presenting a beneficial metabolic profile compared to the MUHO group. MHO is characterised by lower blood pressure, glucose levels and lipid profiles, as well as higher insulin sensitivity, compared to MUHO [20]. In addition, MHO subjects have lower all-cause and cardiovascular disease mortality than MUHO subjects [21]. Therefore, it is important to discriminate the two phenotypes of obesity. However, currently, there is a lack of consensus regarding defining MHO and MUHO subjects [22]. Moreover, factors associated with healthy and unhealthy obesity phenotypes remain unclear [5]. Here, we hypothesis that in addition to lifestyle factors, genetic factors might partly explain the differences between MHO and MUHO subjects. Previously, several studies identified genes which might be potentially associated with different features of the metabolic profile [7,8,9]. Additionally, previous studies suggested that selected polymorphisms in *LTF*, *LRP1*, and *LRP2* genes might be associated with the prevalence of metabolic abnormalities [7,11,12,23,24,25,26].

The *LTF* gene is organised into 17 exons, ranging in size from 23 to 35 kb [27] and is located on human chromosome 3, position 3p2112 [28]. This gene is highly polymorphic with the presence of several common alleles in the general population [27,28]. Previously, several studies have suggested that some SNPs in *LTF* gene might be associated with the prevalence of metabolic abnormalities [11,12,23]; however, none of the studies evaluated the association between *LTF, LRP1* and *LRP2* genes polymorphisms with the prevalence of metabolically healthy or metabolically unhealthy obesity. Similarly to our results, Marcil et al. [12] in a study conducted on 1749 French Canadians aged 9, 13 and 16 years and found a significant difference in allele frequencies between subjects with and without metabolic syndrome for the *LTF* rs2239692 polymorphism. However, the association did not remain significant after correction for multiple testing.

Our study is the first that demonstrated a significant association between the *LTF* rs1126477 gene variants and the anthropometric parameters. More specifically, we noticed that the CT variant of the *LTF* rs1126477 was associated with lower WC in the total population and lower BMI in the MHO group compared to the TT variant. However, our results contrast with those of a previous study conducted on the male population with normal blood glucose levels or an altered glucose tolerance and reported no association between the *LTF* rs1126477 and rs1126478 polymorphisms and the anthropometric parameters. Interestingly, the researchers observed that subjects with a normal glucose tolerance who were AG heterozygotes for *LTF* rs1126477 had significantly decreased TG levels. Similarly, G carriers for *LTF* rs1126478 had significantly lower TG levels and significantly higher HDL-C levels than AA homozygotes. These associations remained significant after controlling for age, BMI, waist-to-hip ratio, fasting glucose concentrations, smoking status, and alcohol intake. In addition, the authors suggested that carriers of the G allele of *LTF* rs1126478 may have a better ability to inhibit modified lipoprotein uptake in macrophages than carriers of the A allele [11]. However, these findings were not confirmed in the present study. Nevertheless, in MHO subjects we found significant differences in LDL-C levels between *LTF* rs1126477 gene variants.

Recently, the association between *LTF* gene polymorphisms and blood pressure was reported, with Alexander et al. [23] observing that *LTF* rs1126478 was over-represented in subjects with hypertension compared to controls. Using a recessive genetic model, researchers found that the frequency of homozygosity for the minor allele (GG) in hypertensive group significantly increased relative to controls. In addition, for an additive genetic model, but not for dominant genetic model, researchers observed a trend for a significant association of *LTF* rs1126478 with hypertension. In our study, for the first time we compared the effect of *LTF, LRP1* and *LRP2* genes polymorphisms on blood pressure in MHO and MUHO groups. We did not find differences between gene variants of *LTF* rs1126478 polymorphism and blood pressure, but we showed significant differences in DBP between *LRP1* rs1799986 gene variants in MUHO subjects.

Regarding putative lactoferrin receptors, LRP1 is an endocytic and signalling receptor which is widely expressed in several tissues. LRP1 is a member of the LDL receptor family which is involved in the clearance of chylomicron remnants from the circulation and present cardioprotective effect. The previous study demonstrated that LRP1 is involved in insulin and glucose homeostasis [29]. Therefore, it was hypothesised that SNPs in the *LRP1* gene might also affect the prevalence of metabolic abnormalities in obese subjects. Indeed, Delgado-Lista et al. [7] evaluated the association of 904 SNPs selected for their potential contribution to carbohydrate metabolism in 450 participants in the LIPGENE cohort and found that fasting insulin, and C-peptide levels, as well as HOMA-IR, and the quantitative insulin sensitivity check index (QUICKI) significantly differed according to *LRP1* rs4759277 gene variants. These results were in contrast to our findings, as we did not observe an association between *LRP1* rs4759277 gene variants and glucose and insulin homeostasis. However, we noted significant differences in HDL-C concentrations between genetic variants of this polymorphism in the MHO group but not in the MUHO group. Besides, we noted that *LRP1* rs4759277 polymorphism is associated with DBP in MUHO and HDL-C levels in MHO subjects. Previously, no studies have reported an association between *LRP1* rs4759277 gene variants and lipid profile as well as blood pressure. Nevertheless, Aledo et al. [24] found that *LRP1* rs1799986 polymorphism in the dominant model (CT + TT vs. CC) was significantly associated with premature cardiovascular disease in familial hypercholesterolemia after adjusting for sex, age and BMI. Besides, Pocathikorn et al. [30] found a significantly lower frequency of TT variant of *LRP1* rs1799986 polymorphism in subjects with coronary heart disease compared to controls. In contrast, Benes et al. [31] showed that subjects with the 5G/5G plasminogen activator inhibitor-1 genotype and the T allele had increased risk of coronary heart disease.

Recent studies have also suggested that *LRP1* is a likely contributor to adipogenesis and adipocyte homeostasis. In addition, it has been shown that the expression of this gene in obese subjects in adipocytes is increased [32,33,34]. Moreover, Hoffman et al. [35] reported that *LRP1* knockout mice have a lower fat mass and elevated energy expenditure, whereas Liu et al. [36] showed that *LRP1* knockout mice have a two-fold increase in fat mass compared to wild-type mice, which was associated with increased food intake, reduced energy expenditure and decreased leptin concentrations. The association of *LRP1* knockout mice with increased fat mass was also supported by Terrand et al. [37], who found that *LRP1* knockout mice had a higher body fat which was associated with reducing lipolysis. Based on these results, we hypothesised that selected SNPs in the *LRP1* gene might be associated with anthropometric parameters. Indeed, Frazier-Wood et al. [38] observed that homozygous subjects for the minor allele at the *LRP1* rs715948 polymorphism had BMIs around 1.03 kg/m^2^ higher than major allele carriers. In our study, we found an association between WC and *LRP1* rs4759277 polymorphism in MHO subjects.

LRP2 encodes low-density lipoprotein receptor-related protein 2 (megalin) and is a member of the low-density lipoprotein receptor family. The receptor is expressed in the epithelial of renal proximal tubules, the epididymis, and thyroid cells and probably play an important role in the reabsorption of proteins and endocytosis [39]. Previously, it was reported that the T allele of *LRP2* rs2544390 polymorphism is associated with higher serum uric acid levels [40,41]. In addition, Nakatochi et al. [25] found that the T allele in *LRP2* rs2544390 polymorphism was significantly associated with a higher risk of metabolic syndrome development in Japanese male employees, whereas Sun et al. [26] noted that the T allele in *LRP2* rs2544390 was significantly correlated with increased fasting insulin concentrations, HOMA-IR and the second-phase Stumvoll index. However, this study was conducted in Chinese women and it cannot be ruled out that in European subjects, other genetic factors affect glucose and insulin homeostasis. Indeed, in our study, we did not observe an association between *LRP2* rs2544390 polymorphism and metabolic abnormalities.

CRP is a biomarker of inflammation which may constitute an independent risk factor for cardiovascular disease [42,43]. In our study, we assessed the impact of analysed genes polymorphisms on CRP levels. However, we did not demonstrate any association. Nevertheless, we showed that in the MHO group statistically significant more subjects had CRP values < 4 mg/L than in the MUHO group (*p* = 0.0120). However, no possibility to obtain high sensitivity CRP values for each included subjects could affect our findings. Our study has some limitations. The small number of tagging SNPs genotyped and the low prevalence of some of the analysed SNPs in the European population could result in limited power to detect significant gene-related associations. Furthermore, it is also possible to obtain false-positive results when several SNPs are analysed. Moreover, our results might be related to other SNPs in linkage disequilibrium with analysed polymorphisms. Furthermore, women were the majority of our population and our analysis was limited to white European descent. Therefore, it is not clear if these results are generalisable to other ethnicities. In addition, a relatively small number of the studied subjects received antihypertensive or hypolipemic drugs and none of the subjects received hypoglycemic treatment. However, the use of antihypertensive, hypolipemic and hypoglycemic therapies is usually common in the obese population. Besides, the MHO group was significantly younger than the MUHO group, so it is also probable that some subjects from the MHO group will develop metabolic abnormalities within a few years. Furthermore, we did not adjust for other confounding factors, such as diet, alcohol consumption and cigarette smoking, as these may bias the association between analysed SNPs and the prevalence of metabolic syndrome. Finally, we did not assess the effect of analysed SNPs on lactoferrin levels.

The strengths of this study are the well-characterised study population and the inclusion of many reliable biochemical parameters. For genotyping, we used TaqMan allelic discrimination assays, the simplest SNPs genotyping technology, which is easy to automate and scale up. Besides, this is the first study that compared the prevalence of selected *LTF* and lactoferrin receptor genes polymorphisms in MHO and MUHO subjects.

## 5. Conclusions

Selected lactoferrin and lactoferrin receptor-related genes variants might be associated with the prevalence of metabolically healthy or metabolically unhealthy phenotypes in obese subjects. However, future studies are needed to understand how the analysed polymorphisms might impact the development of metabolic abnormalities in obese subjects.

## Figures and Tables

**Table 1:** Characteristics of the study population (*n* = 452).

		Median (Q1–Q3)	Mean ± SD
Sex [% of women] ^1^		359 (79.4%)	
Pharmacological treatment [%] ^1,2^		43 (9.5%)	
Age [years]		61 (55–65)	59 ± 10
Body weight [kg]		85.2 (77.6–95.0)	87.4 ± 14.3
Height [cm]		162.0 (157.0–168.0)	162.7 ± 10.0
BMI [kg/m^2^]		31.68 (29.91–35.03)	32.75 ± 4.24
WC [cm]		104.5 (97.0–110.0)	104.9 ± 10.9
TC [mg/dL]		209 (182–239)	213 ± 43
HDL-C [mg/dL]		55 (47–64)	57 ± 14
LDL-C [mg/dL]		119 (95–152)	124 ± 44
TG [mg/dL]		155 (111–215)	180 ± 119
Glucose [mg/dL]		94 (86–105)	99 ± 25
CRP ^1,3^	<4 mg/L	309 (68.7%)	
	≥4 mg/L	141 (31.3%)	
CRP [mg/L] ^4^		4.3 (1.9–7.7)	5.5 ± 4.9
SBP [mmHg]		140 (130–153)	142 ± 17
DBP [mmHg]		85 (78–92)	85 ± 12

^1^*n* (%), ^2^ Antihypertensive drugs: *n* = 41 (9.1%), hypolipemic drugs: *n* = 3 (0.7%), none of the subjects received hypoglycemic drugs, ^3^ *n* = 450, ^4^ *n* = 258.

**Table 2:** Comparison of MHO and MUHO subjects.

		MHO (*n* = 161)		MUHO (*n* = 291)		*p*
		Median (Q1–Q3)	Mean ± SD	Median (Q1–Q3)	Mean ± SD	
Sex [% of women] ^1^		129 (80.1%)		230 (79.0%)		0.7844
Pharmacological treatment [%] ^1,2^		7 (4.3%)		36 (12.4%)		0.0054
Age [years]		60 (53–64)	57 ± 11	62 (56–65)	60 ± 10	0.0102
Body weight [kg]		84.0 (78.1–94.6)	86.5 ± 14.1	86.2 (77.5–95.3)	87.9 ± 14.5	0.4755
Height [cm]		162.0 (158.0–169.0)	162.8 ± 10.5	161.8 (157.0–168.0)	162.7 ± 9.7	0.6512
BMI [kg/m^2^]		31.66 (30.04–34.01)	32.34 ± 3.50	31.77 (29.86–35.36	32.98 ± 4.59	0.4012
WC [cm]		102.0 (96.0–110.0)	102.9 ± 10.7	105.0 (99.0–112.0)	106.0 ± 10.8	0.0112
TC [mg/dL]		202 (181–233)	207 ± 41	216 (183–241)	216 ± 44	0.0463
HDL-C [mg/dL]		60 (54–70)	63 ± 13	53 (45–61)	54 ± 13	<0.0001
LDL-C [mg/dL]		117 (97–148)	122 ± 38	123 (89–153)	126 ± 47	0.5384
TG [mg/dL]		115 (92–141)	122 ± 48	183 (147–243)	213 ± 133	<0.0001
Glucose [mg/dL]		89 (83–95)	89 ± 10	100 (89–112)	105 ± 28	<0.0001
CRP ^1,3^	<4 mg/	121 (76.1%)		188 (64.6%)		0.0120
	≥4 mg/L	38 (23.9%)		103 (35.4%)		
CRP [mg/L] ^4^		3.7 (1.7–6.7)	5.0 ± 5.3	4.6 (2.3–8.1)	5.8 ± 4.7	0.0965
SBP [mmHg]		131 (123–145)	134 ± 17	144 (135–155)	146 ± 16	<0.0001
DBP [mmHg]		81 (73–88)	82 ± 13	87 (80–94)	87 ± 10	<0.0001

^1^*n* (%), ^2^ Including antihypertensive and hypolipemic drugs (none of the subjects received hypoglycemic drugs), ^3^ *n* = 450 (MHO: *n* = 159, MUHO: *n* = 291), ^4^ *n* = 258 (MHO: *n* = 84, MUHO: *n* = 174).

**Table 3:** Distribution of alleles and genotypes in analysed gene polymorphisms in MHO and MUHO subjects.

Polymorphism	Alleles/Genotypes	MHO (*n* = 161)	MUHO (*n* = 291)	*p*
*LTF* rs1126477	C	80.1%	76.1%	0.1669
	T	19.98%	23.9%	
	CC	102 (63.4%)	167 (57.4%)	0.3598
	CT	54 (33.5%)	109 (37.5%)	
	TT	5 (3.1%)	15 (5.1%)	
*LTF* rs1126478	T	73.0%	70.8%	0.4843
	C	27.0%	29.2%	
*LTF* rs1126478	TT	84 (52.2%)	149 (51.2%)	0.4477
	CT	67 (41.6%)	114 (39.2%)	
	CC	10 (6.2%)	28 (9.6%)	
*LTF* rs2239692	T	90.7%	93.3%	0.1561
	C	9.3%	6.7%	
	TT	131 (81.4%)	256 (88.0%)	0.0220
	CT	30 (18.6%)	31 (10.6%)	
	CC	0 (0.0%)	4 (1.4%)	
*LRP1* rs1799986	C	87.6%	85.9%	0.4824
	T	12.4%	14.1%	
	CC	124 (77.0%)	213 (73.2%)	0.5573
	CT	34 (21.1%)	74 (25.4%)	
	TT	3 (1.9%)	4 (1.4%)	
*LPR1* rs4759277	C	59.6%	60.8%	0.7245
	A	40.4%	39.2%	
	CC	58 (36.0%)	109 (37.5%)	0.9409
	CA	76 (47.2%)	136 (46.7%)	
	AA	27 (16.8%)	46 (15.8%)	
*LPR2* rs2544390	C	63.3%	59.5%	0.2495
	T	36.7%	40.5%	
	CC	63 (39.1%)	106 (36.4%)	0.3591
	CT	78 (48.5%)	134 (46.1%)	
	TT	20 (12.4%)	51 (17.5%)	

**Table 4:** Odds ratios (OR) and 95% confidence interval (CI) for the associations between analysed gene polymorphisms and the prevalence of metabolic disorders in obese subjects.

Polymorphism	Allele/Genotypes	OR	95% CI	*p*
*LTF* rs1126477	C ↔ T	1.26	0.91–1.77	0.1668
	CC ↔ CT	1.23	0.82–1.85	0.3151
	CC ↔ TT	1.83	0.65–5.19	0.2484
	TT ↔ CT	0.67	0.23–1.95	0.4629
	CC ↔ CT + TT	1.28	0.86–1.91	0.2160
	CT + CC ↔ TT	0.59	0.21–1.65	0.3104
*LTF* rs1126478	T ↔ C	1.11	0.82–1.51	0.4843
	TT ↔ CT	0.96	0.64–1.44	0.8396
	TT ↔ CC	1.58	0.73–3.41	0.2423
	CC ↔ CT	0.61	0.28–1.33	0.2091
	CC + CT↔ TT	1.04	0.71–1.53	0.8431
	CC ↔ CT + TT	0.622	0.29–1.32	0.2108
*LTF* rs2239692	C ↔ T	1.43	0.87–2.35	0.1561
	CC ↔ CT	0.11	0.01–2.22	0.0560
	CC ↔ TT	0.22	0.01–4.06	0.1536
	TT ↔ CT	0.53	0.31–0.91	0.0204
	CC ↔ CT + TT	0.20	0.01–3.70	0.1351
	CT + CC ↔ TT	0.60	0.35–1.01	0.0553
*LRP1* rs1799986	C ↔ T	1.16	0.77–1.73	0.4825
	CC ↔ CT	1.27	0.80–2.01	0.3152
	CC ↔ TT	0.78	0.17–3.52	0.7422
	TT ↔ CT	1.63	0.35–7.70	0.5324
	CC ↔ CT + TT	1.23	0.78–1.92	0.3715
	CT + CC ↔ TT	1.36	0.30–6.16	0.6869
*LPR1* rs4759277	A ↔ C	1.05	0.80–1.39	0.7244
	AA ↔ AC	1.05	0.60–1.82	0.8615
	AA ↔ CC	1.10	0.62–1.95	0.7367
	CC ↔ AC	0.95	0.62–1.46	0.8211
	AA ↔ AC + CC	1.07	0.64–1.80	0.7900
	AA + AC ↔ CC	0.94	0.63–1.40	0.7626
*LPR2* rs2544390	C ↔ T	1.18	0.89–1.56	0.2580
	CC ↔ CT	1.01	0.66–1.53	0.9784
	CC ↔ TT	1.52	0.83–2.77	0.1757
	TT ↔ CT	0.66	0.37–1.19	0.1702
	CC ↔ CT + TT	1.11	0.75–1.65	0.6066
	CT + CC ↔ TT	0.66	0.38–1.16	0.1450

## Supplementary Materials

The following are available online at https://www.mdpi.com/2072-6643/12/9/2843/s1, Table S1: Characteristics of non-pharmacologically treated subjects. Table S2. Distribution of alleles and genotypes in analysed gene polymorphisms in non-pharmacologically treated subjects. Table S3: Characteristics of the total study population according to *LTF* rs1126477 gene variants. Table S4: Characteristics of MHO subjects according to *LTF* rs1126477 gene variants. Table S5: Characteristics of MUHO subjects according to *LTF* rs1126477 gene variants. Table S6: Characteristics of the total study population according to *LTF* rs1126478 gene variants. Table S7: Characteristics of MHO subjects according to *LTF* rs1126478 gene variants. Table S8: Characteristic of MUHO subjects according to *LTF* rs1126478 gene variants. Table S9: Characteristics of the total study population according to *LTF* rs2239692 gene variants. Table S10: Characteristics of MHO subjects according to *LTF* rs2239692 gene variants. Table S11: Characteristics of MUHO subjects according to *LTF* rs2239692 gene variants. Table S12: Characteristics of the total study population according to *LRP1* rs1799986 gene variants. Table S13: Characteristics of MHO subjects according to *LRP1* rs1799986 gene variants. Table S14: Characteristics of MUHO subjects according to *LRP1* rs1799986 gene variants. Table S15: Characteristics of the total study population according to *LRP1* rs4759277 gene variants. Table S16: Characteristics of MHO subjects according to *LRP1* rs4759277 gene variants. Table S17: Characteristics of MUHO subjects according to *LRP1* rs4759277 gene variants. Table S18: Characteristics of the total study population according to *LRP2* rs2544390 gene variants. Table S19: Characteristics of MHO subjects according to *LRP2* rs2544390 gene variants. Table S20: Characteristics of MUHO subjects according to *LRP2* rs2544390 gene variants. Table S21. Comparison of the number of protective and risk genotypes between MHO and MUHO subjects. Table S22. Characteristics of the study population according to the number of protective genotypes. Table S23. Characteristics of the study population according to the number of risk genotypes.
